# Engagement in gamified virtual reality sports activities and sports participation intention among adolescents with autism spectrum disorder: the mediating roles of immersion and enjoyment

**DOI:** 10.3389/fpubh.2026.1904037

**Published:** 2026-07-20

**Authors:** Ahra Oh, Eun-Hee Park

**Affiliations:** 1 Institute of Sport Science, Korea National Sport University, Seoul, Republic of Korea; 2 Department of Sports Leisure, Sungshin Women's University, Seoul, Republic of Korea

**Keywords:** adolescents, autism spectrum disorder, enjoyment, gamification, immersion, sports participation intention, virtual reality sports

## Abstract

**Background:**

Adolescents with autism spectrum disorder (ASD) face multiple barriers to sports participation because of cognitive rigidity, sensory processing difficulties, and challenges in social interaction. Gamified virtual reality (VR) sports activities have emerged as a potential approach to support physical activity in this population. However, limited research has examined the psychological mechanisms underlying engagement in gamified VR sports activities among adolescents with ASD. Therefore, this study examined the association between engagement in gamified VR sports activities and sports participation intention among adolescents with ASD, focusing on the mediating roles of immersion and enjoyment.

**Methods:**

A total of 179 adolescents with ASD (aged 10–19 years; 78.2% male) and 179 corresponding proxy respondents (teachers and sports instructors) participated in this study, each forming a one-to-one respondent–participant pair. The participants were recruited from special education schools and sports clubs in South Korea that were equipped with non-immersive VR sports systems. Engagement in gamified VR sports activities, immersion, enjoyment, and sports participation intention were assessed using a structured questionnaire. Confirmatory factor analysis was conducted to evaluate the measurement model, and structural equation modeling with bootstrapping was used to test the hypothesized relationships.

**Results:**

Engagement in gamified VR sports activities was positively associated with immersion (*β* = 0.337, *p* < 0.001) and enjoyment (β = 0.435, *p* < 0.001). Both immersion (β = 0.329, *p* < 0.001) and enjoyment (β = 0.403, *p* < 0.001) were significantly associated with sports participation intention. In addition, both immersion (95% CI = 0.155 to 0.382, *p* < 0.001) and enjoyment (95% CI = 0.139 to 0.359, *p* < 0.001) had significant indirect effects on the relationship between engagement and sports participation intention.

**Conclusion:**

The findings suggest that both immersion and enjoyment serve as meaningful psychological mediators linking engagement in gamified VR sports activities to sports participation intention among adolescents with ASD. The structured and predictable nature of gamified VR sports environments may be particularly well-suited to the cognitive and sensory characteristics of adolescents with ASD, facilitating psychological involvement and positive affective responses associated with greater sports participation intention. However, given the cross-sectional design and the use of proxy-reported measures, the findings should be interpreted with caution.

## 1 Introduction

Adolescents with autism spectrum disorder (ASD) face multiple barriers to sports participation because of cognitive rigidity, attention difficulties, and challenges in social interaction and communication. As a result, they often demonstrate lower levels of self-efficacy, motor skills, and physical fitness than their peers without disabilities, which may reduce their engagement in physical activity (1, 2). In addition to individual characteristics, social and environmental barriers—such as a shortage of trained professionals, negative parental perceptions, limited facilities, and transportation constraints—further restrict participation opportunities (3–6). Furthermore, heightened sensitivity to external stimuli and difficulties with sensory processing are particularly pronounced in individuals with ASD and may increase safety concerns, discouraging caregivers and instructors from promoting sports participation (7, 8).

ASD is a neurodevelopmental condition characterized by persistent deficits in social communication and interaction, alongside restricted and repetitive patterns of behavior, interests, and activities (9, 10). These core features often manifest in ways that directly affect sports participation. Specifically, difficulties in understanding social rules, coordinating with peers, and adapting to unpredictable environments can limit opportunities for engagement in organized sports activities (11, 12). In addition, motor coordination difficulties and lower levels of physical fitness are commonly observed in adolescents with ASD, further reducing their confidence and willingness to participate in physical activity (1, 13). Environmental factors, including inadequate facilities, lack of trained professionals, and limited peer support, compound these individual-level barriers and restrict meaningful participation in sports and recreational activities (14, 15).

Virtual reality (VR) technology has emerged as a promising approach in sports, education, and rehabilitation contexts (16–23). In particular, VR-based sports activities provide structured, interactive, and relatively safe environments that may support physical activity participation among adolescents with ASD (24–26). The controlled and predictable nature of VR environments aligns well with the characteristics of ASD, as these environments can offer consistent sensory input, clear task guidance, and immediate feedback, which may help reduce the sensory and social barriers commonly experienced by individuals with ASD (16, 17). Recent studies have further demonstrated that VR-based physical activity interventions can enhance motor performance and enjoyment in adolescents with ASD, suggesting that VR may serve as a particularly suitable medium for promoting physical activity engagement in this population (24, 25).

Within VR environments, psychological experiences play a central role in shaping behavioral outcomes. Among these, immersion and enjoyment have been identified as key factors associated with engagement and participation in physical activity (27–30). Although related, the constructs examined in this study—engagement, immersion, and enjoyment—represent distinct psychological experiences (29, 31, 32). Engagement refers to the degree of behavioral and cognitive involvement in an activity (33), immersion reflects a deeper psychological state characterized by focused attention and reduced awareness of the external environment (31, 34), and enjoyment represents an affective response associated with pleasure and positive feelings during the activity. Enjoyment has also been conceptualized as a positive emotional experience that promotes continued participation (35). While related constructs such as flow and presence are also widely discussed in VR research, flow is typically considered a subjective internal state, whereas presence refers to the sense of “being there” in a virtual environment (31, 36). Given the proxy-based nature of this study and the behavioral observability of attentional responses in adolescents with ASD, immersion was selected as a relatively more observable construct than flow or presence.

Immersion has been shown to enhance attention, involvement, and persistence in VR-based activities, which may contribute to the formation of positive behavioral intentions toward sports participation (29, 37, 38). These attentional and behavioral characteristics of immersion may be particularly relevant for adolescents with ASD, who often exhibit heightened focus on specific stimuli within structured environments. In contrast, enjoyment reflects affective responses that may enhance intrinsic motivation and promote positive exercise experiences (39–41). Although both immersion and enjoyment are important psychological responses, they may operate through distinct mechanisms (34, 36). Immersion is primarily associated with attentional processes and task involvement (42, 43), whereas enjoyment may influence motivation more indirectly through affective pathways (29). Nevertheless, prior research suggests that enjoyment is positively associated with participation intention (40, 44), supporting its inclusion as a potential mediator.

Despite the growing body of research on VR-based physical activity, empirical studies focusing on adolescents with autism spectrum disorder (ASD) remain limited, particularly with respect to gamified VR sports contexts. More importantly, little is known about the psychological mechanisms through which engagement in gamified VR sports activities is associated with sports participation intention in this population, particularly regarding the distinct roles of immersion and enjoyment. Although immersion and enjoyment have been examined in general VR and physical activity research, their mediating roles in the context of gamified VR sports activities among adolescents with ASD have not been sufficiently investigated. Therefore, this study aimed to examine the association between engagement in gamified VR sports activities and sports participation intention among adolescents with ASD, with a particular focus on the mediating roles of immersion and enjoyment. Rather than emphasizing a direct relationship, this study focused on the indirect pathways through which psychological experiences link engagement in VR sports activities to participation intention.

**Figure 1:**
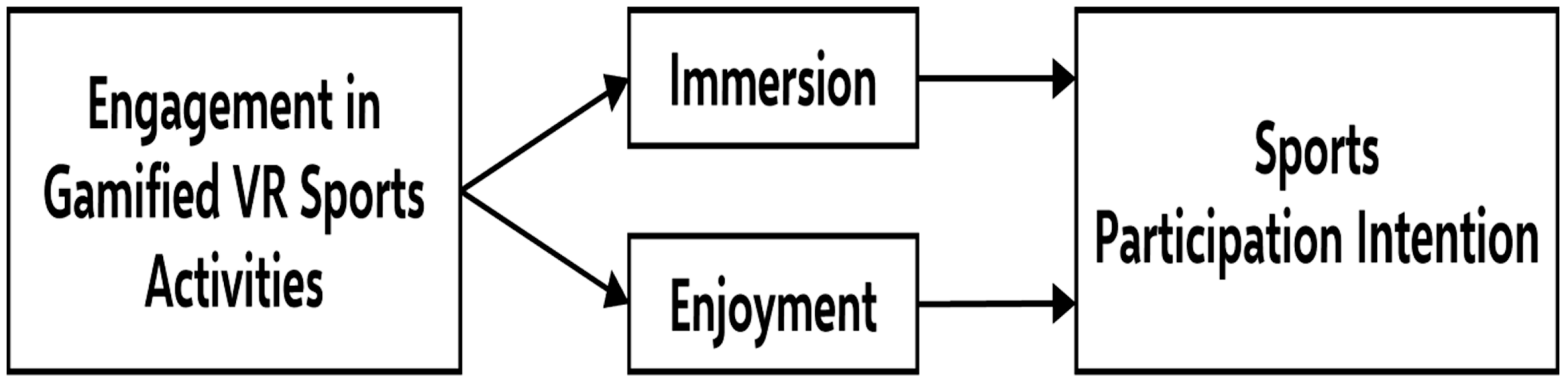
Proposed research model.

Figure 1 presents the proposed research model. Engagement in gamified VR sports activities was specified as the independent variable, immersion and enjoyment as mediating variables, and sports participation intention as the outcome variable. This model was examined among adolescents with autism spectrum disorder (ASD).

## 2 Materials and methods

### 2.1 Participants

Participants in this study were adolescents with autism spectrum disorder (ASD). Data were collected from proxy respondents, including teachers and sports instructors, who regularly interacted with these adolescents during gamified VR sports activities.

Proxy respondents were recruited because adolescents with ASD may experience difficulties with reading, understanding questionnaire items, and recalling information, which may lead to unreliable self-reported data (45). Previous research has also highlighted the limitations of relying solely on self-report measures in this population because of cognitive and communication constraints and has therefore utilized proxy reports from parents or teachers to assess emotional and behavioral characteristics (45–47). In this study, teachers and sports instructors were considered appropriate proxy respondents because they regularly interacted with the youth and could directly observe their behaviors and psychological states. Furthermore, the behavioral and attentional responses of adolescents with ASD are often more readily observable by external raters than those of individuals with other developmental disabilities, which supports the appropriateness of proxy-based assessment in this population. Each proxy respondent was instructed to identify and evaluate one adolescent with mild-to-moderate ASD who was currently engaged in gamified VR sports activities.

The VR sports systems used in this study were screen-based, non-immersive systems installed in special education schools and youth sports facilities for adolescents with disabilities in South Korea. Systems installed in special education schools were procured through a government-led public bidding process, as part of a national initiative to promote physical activity among adolescents with disabilities (48), whereas systems installed in youth sports facilities were independently purchased by each facility. Accordingly, the specific manufacturer varied across institutions. However, all systems shared a common core design consisting of a large wall-mounted projection screen with embedded touch- and motion-sensing technology, allowing participants to interact with the virtual environment using actual sports equipment (e.g., a soccer ball, table tennis paddle, bowling ball, or bow and arrow) rather than a head-mounted display or handheld controller. Physical actions performed by the participant (e.g., kicking, striking, throwing, or shooting) were captured by sensors and translated into corresponding actions within the projected virtual environment. The systems supported multiple sports activities, including table tennis, archery, basketball, bowling, and soccer, and incorporated gamification elements such as real-time scoring, visual and auditory feedback, progress indicators, and multiplayer participation. Although the specific configuration of available sports and equipment varied slightly across institutions depending on the installed system, all systems shared the same core screen-based, non-immersive, sensor-driven design.

Each proxy respondent was paired with one adolescent participant, resulting in 179 respondent–participant dyads. A total of 179 proxy respondents were recruited using convenience sampling. Although this sample size is slightly below the commonly recommended minimum of 200 for SEM (49), it exceeds the threshold of 150 participants suggested for models with more than ten measurement variables (50). Moreover, the use of a diagnostically homogeneous sample of adolescents with ASD, rather than a broader developmental disability group, may enhance the internal validity of the findings.

The study protocol was approved by the Institutional Review Board of Korea National Sport University, Seoul, Republic of Korea (KNSU IRB 20221209-095; December 9, 2022). All procedures were conducted in accordance with the Declaration of Helsinki. Prior to participation, adolescents with ASD and their parents or legal guardians received a detailed explanation of the study purpose and procedures, and written informed consent was obtained. In addition, proxy respondents (teachers and instructors) who provided the data also received a full explanation of the study and provided written informed consent prior to participation.

This study was conducted and reported in accordance with the Strengthening the Reporting of Observational Studies in Epidemiology (STROBE) guidelines. Table 1 presents the characteristics of the participants and proxy respondents.

**Table 1:** Characteristics of the participants and proxy respondents.

Characteristic		Frequency	Percentage
Sex of proxy respondents	Male	124	69.3
	Female	55	30.7
Sex of participants	Male	140	78.2
	Female	39	21.8
Age of proxy respondents	20–29	19	10.6
	30–39	81	45.3
	40–49	68	38.0
	50–59	11	6.1
Age of participants	10–14	77	43.0
	15–19	102	57.0
Disability severity level of participants	Mild	59	33.0
	Moderate	120	67.0
Frequency of engagement in VR-based activities	Once per week	89	49.7
	Twice per week	76	42.5
	Three times per week	10	5.6
	More than three times per week	4	2.2

VR, virtual reality.

We acknowledge the ongoing terminological debate regarding the use of person-first language (e.g., “adolescents with ASD”) versus identity-first language (e.g., “autistic adolescents”). Although a growing body of guidance from autistic self-advocacy organizations favors identity-first language, person-first language remains widely used in clinical, educational, and special physical activity research in South Korea, where this study was conducted. We adopted person-first language throughout this manuscript for consistency with this research tradition, while recognizing that identity-first language may be preferred by some members of the autistic community.

### 2.2 Measurement instrument

Engagement in gamified VR sports activities, immersion, enjoyment, and sports participation intention were measured using a structured questionnaire. The questionnaire was adapted from previously validated instruments, with minor modifications to fit the context of gamified VR sports activities and to enable proxy reporting by teachers and sports instructors. All items were derived from established scales reported in prior studies, and no entirely new measurement instrument was developed.

Given that teachers and sports instructors provided information on behalf of the participants, the item wording was modified to operationalize the psychological constructs through observable behaviors and responses during VR sports activities. Specifically, first-person expressions, such as “I,” in the original self-report items were replaced with references to “adolescents with ASD,” and subjective statements, such as “I was focused,” were reformulated into observable behavioral descriptions, such as “the adolescent appears to be deeply engaged in the activity through sustained attention.”

To ensure the semantic and conceptual equivalence of the adapted items, a content validity evaluation was conducted. The revised items were reviewed by two experts with doctoral degrees in adapted physical activity and sports for individuals with disabilities and one expert with a doctoral degree in sport psychology. Based on their feedback, the items were refined to ensure clarity, relevance, and consistency with the original constructs.

In general, flow is used to measure high levels of concentration and a sense of control during VR activities. However, flow is a subjective psychological state typically assessed through self-report measures (46). Given that the participants in this study were adolescents with ASD, self-reporting was not feasible due to the cognitive and communication characteristics associated with ASD. Therefore, immersion—a construct that can be partially inferred from observable behavioral engagement—was considered more appropriate than flow (18, 51).

It is important to clarify the conceptual scope of immersion as operationalized in this study. Immersion in VR research has been conceptualized along both technological (e.g., perceptual fidelity, field of view, sensory bandwidth) and psychological dimensions (e.g., attentional absorption, reduced awareness of the external environment) (31, 34). The present study focused exclusively on the psychological dimension of immersion, independent of the technological immersion’s level of the display system. Notably, the immersion framework adopted in this study (42) was originally developed and validated in the context of conventional, non-immersive, screen-based video games rather than head-mounted-display VR systems and explicitly conceptualizes immersion as a subjective state of attentional absorption rather than a property of the display hardware. This precedent supports the measurement of psychological immersion independently of hardware-related immersion. Within this framework, immersion was operationally distinguished from engagement: while engagement captured the adolescent’s behavioral and cognitive involvement in the task (e.g., active interaction, task performance), immersion captured the depth of attentional absorption during that involvement (e.g., sustained focus, reduced responsiveness to external stimuli). This conceptual separation allowed the two constructs to be measured as empirically distinguishable, despite both being assessed within a non-immersive technological environment.

Items measuring engagement in gamified VR sports activities were adapted from the Virtual Experience Test developed by Chertoff et al. (47), which has been widely used in VR research (52, 53). Four items were modified to reflect the context of gamified VR sports activities.

Immersion was measured based on the conceptual framework proposed by Jennett et al. (42), with three items adapted from previous studies on VR participation and immersion (54, 55). Enjoyment was assessed using three items adapted from prior studies examining VR participation and enjoyment (55, 56), with wording modifications to allow proxy respondents to evaluate participants’ emotional responses during VR activities. Sports participation intention was measured using two items adapted from studies on VR participation intention (54, 57, 58), modified to fit the context of adolescents with ASD. Overall, the questionnaire consisted of 12 items, all rated on a five-point Likert scale ranging from 1 (“strongly disagree”) to 5 (“strongly agree”). The sources of all the measurement items were cited.

### 2.3 Data analysis

As this study employed a cross-sectional design, the data were analyzed using IBM SPSS Statistics and AMOS 31.0 (IBM Corp., Armonk, NY, United States). Before the main analyses, the data were screened for missing values, normality, and outliers. Missing data accounted for less than 2% of the total responses and were handled using listwise deletion. Normality was assessed using skewness and kurtosis, with cutoff values of ± 2.00 and ± 7.00, respectively, based on the criteria suggested by Kline (49). Multivariate outliers were examined using Mahalanobis distance at *p* < 0.001, and no significant outliers were identified.

First, frequency analysis was performed to examine the characteristics of the participants and proxy respondents. Second, Pearson’s correlation analysis was conducted to examine relationships among the variables and assess multicollinearity among the constructs. Third, a reliability analysis and a confirmatory factor analysis (CFA) were conducted to evaluate the reliability and validity of the measurement instrument. Model fit was assessed using several fit indices. According to commonly accepted criteria, comparative fit index (CFI) and Tucker–Lewis index (TLI) values greater than 0.90 and a root mean square error of approximation (RMSEA) value less than 0.08 were deemed to indicate acceptable model fit (59). In addition, supplementary diagnostic analyses were conducted to examine potential common method variance and multicollinearity. Harman’s single-factor test and variance inflation factor (VIF) analysis and tolerance statistics were performed as additional diagnostic procedures. Finally, SEM was performed to test the proposed research model and hypotheses. In addition, a bootstrapping analysis with 5,000 resamples and a 95% bias-corrected confidence interval was conducted to test the mediating effects (59). The significance of indirect effects was determined using bias-corrected bootstrap confidence intervals.

To empirically justify the full mediation model specified in this study, an alternative partial mediation model—including a direct path from engagement in gamified VR sports activities to sports participation intention—was also estimated. The two models were compared using a chi-square difference test (Δχ^2^) and the full mediation model was retained if the addition of the direct path did not significantly improve model fit.

## 3 Results

### 3.1 Correlation analysis

Table 2 presents the results of Pearson’s correlation analysis. Engagement in gamified VR sports activities was positively correlated with immersion (r = 0.288, *p* < 0.001), enjoyment (r = 0.335, *p* < 0.001), and sports participation intention (r = 0.323, *p* < 0.001). Immersion was significantly positively correlated with enjoyment (r = 0.450, *p* < 0.001) and sports participation intention (r = 0.477, *p* < 0.001). Similarly, enjoyment was positively correlated with sports participation intention (r = 0.529, *p* < 0.001). The correlations among all constructs were moderate, suggesting acceptable discriminant validity between the constructs.

**Table 2:** Results of Pearson’s correlation analysis.

Variable	1	2	3	4
1. Engagement in gamified VR sports activities	1.000	0.288***	0.335***	0.323***
2. Immersion	0.288***	1.000	0.450***	0.477***
3. Enjoyment	0.335***	0.450***	1.000	0.529***
4. Sports participation intention	0.323***	0.477***	0.529***	1.000

VR = virtual reality; ***p < 0.001, two-tailed.

### 3.2 Analysis of the measurement model

A CFA was conducted to examine the construct validity of the measurement model. The model fit indices were as follows: χ^2^ = 108.027 (*df* = 48, *p* < 0.001), TLI = 0.939, CFI = 0.956, and RMSEA = 0.079. Although the TLI (0.939) was slightly below the conventional cutoff of 0.95, both the CFI (0.956) and RMSEA (0.079) met the recommended criteria, indicating acceptable model fit (59).

Next, construct reliability (CR) was examined to assess the reliability of the constructs, and AVE values were used to evaluate convergent validity. The CR values were greater than 0.850, and the AVE values were greater than 0.657, thereby exceeding the recommended thresholds of 0.70 and 0.50, respectively (50). Therefore, the measurement model demonstrated satisfactory convergent validity and reliability.

Cronbach’s alpha coefficients were calculated to assess the internal consistency of the measurement items. All coefficients exceeded the recommended threshold of 0.70, indicating acceptable internal consistency (49). Finally, discriminant validity was examined by determining whether the square root of the AVE for each construct exceeded the correlations among the constructs (50). Table 3 presents the results of the reliability and validity analyses.

**Table 3:** Results of the measurement model analysis.

Factor	Items		Standard loading	CR	AVE	α
Engagement in gamified VR sports activities	1	Adolescents with ASD actively interact with computer agents in the virtual environment.	0.847	0.918	0.737	0.880
	2	Adolescents with ASD are able to perform the tasks presented in the VR sports activity.	0.862			
	3	Adolescents with ASD actively participate in the VR sports activity.	0.776			
	4	The virtual environment helps adolescents with ASD understand the tasks that they must perform.	0.872			
Immersion	5	During the activity, adolescents with ASD demonstrate sustained concentration on the task, showing minimal responsiveness to environmental or external stimuli.	0.789	0.850	0.657	0.840
	6	Adolescents with ASD appear deeply immersed in the activity, as evidenced by continuous movements and a focused gaze.	0.766			
	7	Adolescents with ASD demonstrate sustained attention on the activity, with little distraction from other things.	0.804			
Enjoyment	8	Adolescents with ASD appear to have fun while engaging in the activity.	0.875	0.864	0.680	0.862
	9	Adolescents with ASD appear to enjoy participating in the activity.	0.842			
	10	Adolescents with ASD appear to find the activity interesting.	0.792			
Sports participation intention	11	Adolescents with ASD want to participate in VR sports activities.	0.884	0.867	0.766	0.851
	12	Adolescents with ASD want to participate in VR sports regularly.	0.806			
χ^2^ = 108.027 (*df* = 48, *p* < 0.001), TLI = 0.939, CFI = 0.956, RMSEA = 0.079						

CR, construct reliability; AVE, average variance extracted; VR, virtual reality; TLI, Tucker–Lewis index; CFI, comparative fit index; RMSEA, root mean square error of approximation.

To further examine potential concerns regarding common method variance and multicollinearity, additional diagnostic analyses were conducted, including Harman’s single-factor test and VIF analysis in Table 4. Harman’s single-factor test indicated that the first factor accounted for 42.08% of the total variance, which is below the commonly used threshold of 50%, suggesting that common method variance was not a serious concern in the present study (59). The VIF values ranged from 1.799 to 3.576, while the tolerance values ranged from 0.280 to 0.556, indicating that multicollinearity was not a serious concern (59).

**Table 4:** Additional diagnostic analyses for common method variance and multicollinearity.

Analysis	Result	Reference criterion	Interpretation
Harman single-factor test	42.08%	< 50% preferred	Common method variance not a serious concern
Variance inflation factor	1.799–3.576	< 5 acceptable	Moderate but acceptable
Tolerance	0.280–0.556	> 0.200 acceptable	Acceptable

### 3.3 Analysis of the structural model

Structural equation modeling (SEM) was conducted using the maximum likelihood estimation method to examine the relationships among the variables. The proposed model demonstrated an acceptable overall fit to the data (χ^2^ = 135.183, *df* = 50, *p* < 0.001, CFI = 0.937, TLI = 0.917, RMSEA = 0.093). Although the RMSEA value slightly exceeded the commonly recommended threshold of 0.080, other fit indices, namely CFI and TLI, indicated acceptable model fit (59).

Modification indices were also reviewed to determine whether the marginal RMSEA value could be improved through theoretically justifiable adjustments. The largest indices were associated with the residual covariance and cross-loading paths between immersion and enjoyment (M. I. = 24.10, 20.76, and 18.52, respectively).

The results showed that engagement in gamified VR sports activities was significantly and positively associated with immersion (*β* = 0.337, *p* < 0.001) and enjoyment (β = 0.435, *p* < 0.001). Immersion was significantly and positively associated with sports participation intention (β = 0.329, *p* < 0.001), and enjoyment was also significantly and positively associated with sports participation intention (β = 0.403, *p* < 0.001). All four hypothesized paths were statistically significant, supporting the proposed relationships (see Table 5).

**Table 5:** Results of path analysis.

Predictor → Outcome	Estimate	t-value	*p*
Engagement in gamified VR sports activities → Immersion	0.337	4.282	<0.001
Engagement in gamified VR sports activities → Enjoyment	0.435	5.562	<0.001
Immersion → Sports participation intention	0.329	4.435	<0.001
Enjoyment → Sports participation intention	0.403	5.322	<0.001

VR, virtual reality. All paths were statistically significant at *p* < 0.001.

To empirically evaluate whether full mediation was an appropriate representation of the data, an alternative partial mediation model was estimated by adding a direct path from engagement in gamified VR sports activities to sports participation intention. The partial mediation model showed comparable fit (χ^2^ = 134.737, *df* = 49, CFI = 0.937, TLI = 0.915, RMSEA = 0.094), and the additional direct path was not statistically significant (*β* = 0.055, *p* = 0.518). A chi-square difference test comparing the full mediation model and the partial mediation model indicated no statistically significant improvement in model fit (Δχ^2^ = 0.446, Δ*df* = 1, *p* > 0.05). In addition, the full mediation model showed a lower AIC value than the partial mediation model (AIC = 215.183 vs. 216.737), indicating better relative fit. Based on these results, the full mediation model was retained as the final model, as it provided an equally adequate and more parsimonious representation of the data (see Table 6).

**Table 6:** Comparison of full and partial mediation models.

Model	χ^2^	*df*	AIC	CFI	TLI	RMSEA	Direct path (engagement → SPI)
Full mediation (M1)	135.183	50	215.183	0.937	0.917	0.093	—
Partial mediation (M2)	134.737	49	216.737	0.937	0.915	0.094	β = 0.055, *p* = 0.518

SPI, sports participation intention; AIC, Akaike information criterion; CFI, comparative fit index; TLI, Tucker–Lewis index; RMSEA, root mean square error of approximation. Δχ^2^ = 0.446, Δdf = 1, *p* > 0.05, indicating no statistically significant improvement in model fit with the addition of the direct path. The full mediation model (M1) was retained as the final model based on its lower AIC and more parsimonious structure.

A bootstrapping analysis was conducted to examine the mediating roles of immersion and enjoyment in the relationship between engagement in gamified VR sports activities and sports participation intention. Indirect effects were considered statistically significant when the 95% confidence interval did not include zero (59). The results showed that engagement in gamified VR sports activities had a significant indirect effect on sports participation intention through immersion (95% CI = 0.155 to 0.382, *p* < 0.001), as the confidence interval did not include zero. This finding supports the mediating role of immersion in the relationship between engagement and sports participation intention.

Furthermore, the indirect effect of engagement on sports participation intention through enjoyment was also statistically significant (95% CI = 0.139 to 0.359, *p* < 0.001), as the confidence interval did not include zero. Therefore, the mediating role of enjoyment in the relationship between engagement and sports participation intention was supported.

The total indirect effect of engagement in gamified VR sports activities on sports participation intention was significant (95% CI = 0.178 to 0.418, *p* < 0.001), indicating that the relationship between engagement and sports participation intention was explained through the mediating pathways of both immersion and enjoyment (see Table 7).

**Table 7:** Specific indirect effects of mediation pathways.

Indirect pathway	95% CI
Engagement in gamified VR sports activities → Immersion → Sports participation intention	0.155 to 0.382
Engagement in gamified VR sports activities → Enjoyment → Sports participation intention	0.139 to 0.359

CI = confidence interval; VR = virtual reality. Bias-corrected 95% confidence intervals were obtained from 5,000 bootstrap samples. All indirect effects were statistically significant at *p* < 0.001.

## 4 Discussion

This study examined the relationships among engagement in gamified VR sports activities, immersion, enjoyment, and sports participation intention in adolescents with autism spectrum disorder (ASD). The results showed that engagement was positively associated with both immersion and enjoyment. Immersion was positively associated with sports participation intention and mediated the relationship between engagement and intention. Enjoyment was also positively associated with sports participation intention and mediated the relationship between engagement and intention. These findings suggest that both immersion and enjoyment serve as meaningful psychological pathways linking engagement in gamified VR sports activities to sports participation intention among adolescents with ASD.

First, the positive associations of engagement with both immersion and enjoyment suggest that gamified VR sports activities may enhance psychological involvement and positive affective responses among adolescents with ASD. This finding is consistent with prior research indicating that structured environments, immediate feedback, and game-based elements in VR can facilitate psychological engagement (29, 43, 60, 61). For adolescents with ASD, clearly defined tasks, predictable rules, and visual cues may play a particularly critical role in promoting attention and sustained participation (62–64). The structured and rule-based nature of gamified VR sports environments may align well with the cognitive and behavioral characteristics of ASD, as adolescents with ASD often respond positively to predictable, low-ambiguity environments that minimize unexpected social demands. This alignment may partly explain why engagement in such environments was associated with both immersion and enjoyment in the present study.

Second, both immersion and enjoyment were significantly associated with sports participation intention and mediated the relationship between engagement and intention. This finding suggests that gamified VR sports activities may activate distinct but complementary psychological pathways among adolescents with ASD. Immersion is closely related to attentional focus and behavioral engagement, which may be more directly linked to participation intention (65, 66). Adolescents with ASD often demonstrate heightened focus on specific stimuli within structured environments, and the rule-based, visually engaging nature of gamified VR sports may facilitate sustained attentional involvement, thereby contributing to stronger participation intention (62, 63). The significant mediating role of immersion in the present study is consistent with prior research suggesting that immersive experiences in VR environments can strengthen behavioral intentions toward physical activity (37, 38).

The significant mediating role of enjoyment is a notable finding that distinguishes the present study from prior research conducted with broader developmental disability populations. Enjoyment, as a positive affective response, may play a particularly meaningful role among adolescents with ASD in the context of gamified VR sports activities. Prior research has suggested that adolescents with ASD may respond positively to structured, low-pressure activity environments that minimize social demands and provide consistent sensory feedback (13, 14). The gamified VR sports context may create such conditions, potentially enabling adolescents with ASD to experience and express enjoyment more readily than in conventional sports settings. Furthermore, the behavioral observability of enjoyment-related responses—such as smiling, increased engagement, and vocalization—may have facilitated more accurate proxy assessment in this population, potentially strengthening the observed relationship between enjoyment and participation intention (45). Furthermore, comparison with an alternative partial mediation model—which included a direct path from engagement to sports participation intention—indicated that this direct path was not statistically significant (*β* = 0.055, *p* = 0.518) and did not significantly improve model fit relative to the full mediation model (Δχ^2^ = 0.446, Δdf = 1, *p* > 0.05; AIC = 215.183 vs. 216.737). This result suggests that, within the present sample, the association between engagement and sports participation intention was fully explained through immersion and enjoyment, providing empirical support for the proposed full mediation model.

These findings also contribute to a broader theoretical understanding of how gamified technology-based interventions promote engagement and participation among individuals with disabilities. Self-determination theory posits that motivation and sustained engagement are fostered when activities support basic psychological needs, particularly competence and intrinsic enjoyment (39). Within this framework, immersion may be understood as reflecting need-supportive attentional engagement with the task itself, whereas enjoyment may reflect the affective fulfillment associated with intrinsically motivating experiences. The present findings extend this framework by demonstrating that, among adolescents with ASD, both pathways operate in parallel rather than redundantly, suggesting that gamified VR sports activities may simultaneously facilitate attentional and affective need-fulfillment mechanisms in this population. This pattern is broadly consistent with recent findings in educational technology research, where gamified, technology-assisted interventions incorporating structured feedback and motivational design elements have been shown to enhance both behavioral engagement and psychological need satisfaction in physical skill learning contexts (67, 68). Taken together, these findings suggest that the theoretical mechanisms underlying gamified technology-assisted engagement may generalize, at least in part, across populations and technological platforms, while also highlighting population-specific considerations—such as sensory accessibility and proxy-based assessment—that are particularly salient for adolescents with ASD.

Beyond their theoretical contributions, these findings carry several practical implications. For special education practitioners and sports instructors, the results suggest that gamified VR sports programs incorporating clear task structure, predictable rules, and consistent visual and auditory feedback may be particularly effective in promoting both attentional engagement and positive affective experiences among adolescents with ASD (62, 69). Practitioners may consider prioritizing VR sports activities that offer structured progression and immediate feedback, as these features appear to align well with the cognitive and sensory characteristics commonly observed in this population. For program designers, the findings underscore the value of incorporating both task-focused (immersion-supporting) and affect rewarding (enjoyment-supporting) elements into VR sports content, rather than optimizing for either dimension alone, consistent with prior design guidance for VR interventions for individuals with intellectual and developmental disabilities (64). For policymakers, given that the VR systems used in this study were distributed in part through a government-led initiative (48), these findings provide preliminary evidence supporting the continued expansion of screen-based, non-immersive VR sports infrastructure in special education and community sports settings as a scalable and accessible approach to promoting intentions to participate in physical activity among adolescents with ASD (3). Future investment decisions may benefit from considering not only the availability of VR systems but also the specific gamification features that appear to support psychological engagement in this population.

Several methodological considerations should be noted when interpreting these findings. First, the present study focused exclusively on adolescents with ASD. Although the sample included both mild (33.0%) and moderate (67.0%) levels of ASD severity, sample size limitations precluded subgroup analyses examining whether the mediating roles of immersion and enjoyment differed by severity level, age group, or cognitive ability. Future research with larger samples should prioritize this line of inquiry. Second, as this study employed a cross-sectional design, mediation effects should be interpreted as associations rather than causal relationships, and temporal ordering among the constructs cannot be confirmed (70, 71). Third, the use of proxy-based assessment from a single source at a single time point may have introduced common method bias. Although supplementary diagnostic analyses suggested that this concern was unlikely to substantially affect the findings (Harman’s single-factor test = 42.08%, below the 50% threshold; VIF = 1.799–3.576), it cannot be completely ruled out. Relatedly, the structural model showed a marginal RMSEA value (0.093), slightly exceeding the conventional threshold of 0.080. Modification indices indicated that the largest potential improvement in fit would involve a residual covariance between immersion and enjoyment (M.I. = 24.10). However, this modification was not implemented, as it was not theoretically specified in the proposed model and would risk capitalizing on chance characteristics of the sample (72). The marginal RMSEA value may, therefore, partly reflect unmodeled shared variance between these two constructs, which is consistent with their moderate observed correlation (r = 0.450), and the path estimates should be interpreted accordingly. Fourth, sports participation intention, rather than actual behavior, was used as the outcome variable. Although intention is a key predictor of behavior (73), an intention–behavior gap has been well documented (74, 75). This distinction is particularly important for adolescents with ASD, for whom contextual factors, such as family support, the sensory accessibility of physical environments, and the availability of adapted sports programs, may moderate whether participation intention translates into actual behavior (7, 8, 11, 13, 14, 17). Future research should incorporate longitudinal designs, multi-source measurement, and objective measures of physical activity participation to address these limitations. In summary, engagement in gamified VR sports activities was positively associated with sports participation intention through the mediating roles of immersion and enjoyment among adolescents with ASD. These findings suggest that gamified VR sports environments may support both attentional and affective processes relevant to sports participation in this population. Despite the methodological limitations noted above, the study provides preliminary evidence regarding the psychological mechanisms through which VR sports experiences may promote sports participation intention among adolescents with ASD.

## 5 Conclusion

Engagement in gamified VR sports activities was positively associated with immersion and enjoyment among adolescents with ASD. Both immersion and enjoyment were significantly associated with sports participation intention and mediated the relationship between engagement and participation intention. These findings suggest that gamified VR sports activities may activate distinct but complementary psychological pathways—attentional involvement through immersion and positive affective responses through enjoyment—that together contribute to stronger sports participation intention among adolescents with ASD.

These findings suggest that experience-related factors, particularly immersion and enjoyment, may be associated with sports participation intention among adolescents with ASD. The structured, predictable, and visually engaging nature of gamified VR sports environments may be particularly well-suited to the cognitive and sensory characteristics of this population, facilitating both psychological involvement and positive affective responses. In particular, the gamified VR sports context may create low-pressure, socially undemanding conditions that enable adolescents with ASD to experience and express enjoyment more readily than in conventional sports settings.

Overall, the findings provide preliminary evidence regarding the potential role of VR-based physical activity programs in promoting sports participation intention among adolescents with ASD. Given the limitations of the present study, including its cross-sectional design, reliance on proxy measures, and use of a non-immersive VR environment, the results should be interpreted with caution.

Future research should further examine the relationship between VR sports engagement and actual participation behavior by incorporating diverse and fully immersive VR environments, employing longitudinal designs, and adopting multimethod measurement approaches. Where feasible, self-report measures from adolescents with ASD should be incorporated alongside proxy reports to provide a more comprehensive understanding of their psychological experiences during VR sports activities.

## Data Availability

The datasets presented in this article are not readily available because the data presented in this study are available on request from the first author. Some information cannot be shared to preserve the anonymity of the participants. Requests to access the datasets should be directed to Ahra Oh, oh-yang0329@hanmail.net.
